# The Emergence of Invasive *Streptococcus pneumoniae* Serotype 24F in Lebanon: Complete Genome Sequencing Reveals High Virulence and Antimicrobial Resistance Characteristics

**DOI:** 10.3389/fmicb.2021.637813

**Published:** 2021-02-19

**Authors:** Lina Reslan, Marc Finianos, Ibrahim Bitar, Mohamad Bahij Moumneh, George F. Araj, Alissar Zaghlout, Celina Boutros, Tamima Jisr, Malak Nabulsi, Gilbert Kara yaccoub, Monzer Hamze, Marwan Osman, Elie Bou Raad, Jaroslav Hrabak, Ghassan M. Matar, Ghassan Dbaibo

**Affiliations:** ^1^Center for Infectious Diseases Research (CIDR) and WHO Collaborating Center for Reference and Research on Bacterial Pathogens, American University of Beirut, Beirut, Lebanon; ^2^Department of Microbiology, Faculty of Medicine and University Hospital in Plzen, Charles University, Plzen, Czechia; ^3^Department of Pathology and Laboratory Medicine, American University of Beirut Medical Center, Beirut, Lebanon; ^4^Department of Laboratory and Blood, Makassed General Hospital, Beirut, Lebanon; ^5^Laboratory, Haykal Hospital, Tripoli, Lebanon; ^6^Department of Microbiology, Nini Hospital, Tripoli, Lebanon; ^7^El-Youssef Hospital Center, Department of Clinical Laboratory, Halba, Lebanon; ^8^Department of Experimental Pathology, Immunology, and Microbiology, Faculty of Medicine, American University of Beirut, Beirut, Lebanon; ^9^Department of Pediatrics and Adolescent Medicine, Faculty of Medicine, American University of Beirut, Beirut, Lebanon

**Keywords:** *Streptococcus pneumoniae*, serotype 24F, whole-genome sequencing, Lebanon, antibiotic resistance

## Abstract

**Background:**

Invasive pneumococcal disease (IPD) remains a global health problem. IPD incidence has significantly decreased by the use of pneumococcal conjugate vaccines (PCV). Nevertheless, non-PCV serotypes remain a matter of concern. Eight *Streptococcus pneumoniae* serotype 24F isolates, belonging to a non-PCV serotype, were detected through the Lebanese Inter-Hospital Pneumococcal Surveillance Program. The aim of the study is to characterize phenotypic and genomic features of the 24F isolates in Lebanon.

**Methods:**

WGS using long reads sequencing (PacBio) was performed to produce complete circular genomes and to determine clonality, antimicrobial resistance and virulence determinants.

**Results:**

The sequencing results yielded eight closed circular genomes. Three multilocus sequence typing (MLST) types were identified (ST11618, ST14184, ST15253). Both MLST and WGS analyses revealed that these isolates from Lebanon were genetically homogenous belonging to clonal complex CC230 and clustered closely with isolates originating from Canada, United States of America, United Kingdom and Iceland. Their penicillin binding protein profiles correlated with both β-lactam susceptibility patterns and MLST types. Moreover, the isolates harbored the macrolide and tetracycline resistance genes and showed a similar virulence gene profile. To our knowledge, this study represents the first report of complete phenotypic and genomic characterization of the emerging *Streptococcus pneumoniae*, serotype 24F, in the Middle East and North Africa region.

## Introduction

*Streptococcus pneumoniae* is a major causative agent responsible for severe infections mainly among young children, elderly individuals and those with chronic illnesses and immunodeficiency disorders (Global Burden of Disease Study, 2018; Principi et al., 2018). The World Health Organization (WHO) estimated that pneumococcal disease is responsible for 1.6 million deaths every year, with 0.7 million to 1 million being children younger than 5 years, mostly in developing countries (World Health Organization, 2007).

Based on the capsular polysaccharide structure, almost 100 pneumococcal serotypes have been identified so far (Ganaie et al., 2020). These serotypes vary in terms of incidence, antibiotic resistance, and disease manifestation (Hausdorff et al., 2005) and only a limited number of them cause the majority of invasive pneumococcal disease (IPD) (Namkoong et al., 2016). Introduced for adults in 1983, the 23-valent pneumococcal polysaccharide vaccine (PPV23), targeting 23 serotypes represents 85–90% of all serotypes causing IPD (Pitsiou and Kioumis, 2011). The pneumococcal conjugate vaccine (PCV) that covers seven serotypes also known as PCV7 (4, 6B, 9 V, 14, 18C, 19F, and 23F) was implemented as a routine vaccination in 2000 and was replaced by PCV10 with three additional serotypes (1, 5, and 7F) followed by PCV13 covering six additional serotypes from PCV7 (1, 5, 7F, 3, 6A, and 19A). Those vaccines are recommended for children under the age of 2 (Daniels et al., 2016; Isturiz et al., 2018). The pediatric use of PCV13 has led to sharp declines in pneumococcal disease among unvaccinated adults and children, which led the Advisory Committee on Immunization Practices (ACIP) to reevaluate its use in the United States for adults and recommend its administration for adults aged ≥ 65 years with only underlying medical conditions (Matanock et al., 2019). In Lebanon, PCV7 was introduced by the private sector, in 2006, followed by PCV10 and PCV13 in 2010. As of January 2016, PCV13 has been added to the expanded program of Immunization using the 2++1 schedule. However, there are no official national recommendations for adult immunization, and limited only to some practitioners following the international guidelines in their private practice.

The PCVs introduced has proven to be very effective against vaccine serotypes causing IPD. Yet, this reduction is partly offset by an increase in non-vaccine serotypes (NVT), known as serotype replacement that might be occurring through mainly two mechanisms (Weinberger et al., 2011). The first is expansion of non-vaccine-type lineages to partly occupy the niche vacated by vaccine-type lineages and the second is the expansion of preexisting clones of non-vaccine serotypes within the same lineage to replace vaccine-type serotypes after PCV13 introduction (Croucher et al., 2013). The level of antibiotic consumption undoubtedly plays a role in inducing a selective pressure on pneumococcal strains from nasopharyngeal microbiota and certainly influences serotype distribution in different countries (Lo et al., 2019)Additionally, elevated recombination rates in this species within its locus may drive the change of serotype through a process known as “serotype switching”(Ansaldi et al., 2011) for instance serotype 19A donors and recipients belonging to serotype 4 (Hicks et al., 2007) or serotype 9V and 6A (Vestrheim et al., 2012). Furthermore, vaccine schedules and vaccine coverage can also affect the evolution of pneumococcal epidemiology.

Most of the published data about pneumococcal disease from the Middle East and North Africa (MENA), including Lebanon, focused on the incidence of IPD, the serotypes distribution, and the vaccine coverage (Hanna-Wakim et al., 2012; Mokaddas and Albert, 2012; Taha et al., 2012; Al-Sheikh et al., 2014; Bahy et al., 2016; Moghnieh et al., 2018, 2020). In Lebanon, the Lebanese Inter-hospital Pneumococcal Surveillance program (LIPSP), serves as a national prospective surveillance program, established in 2005 in collaboration with the Lebanese Ministry of Public Health. Previously, we reported data from LIPSP, collected from October 2005 till December 2011, and identified a total of 257 isolates of invasive *S. pneumoniae.* The vaccine coverage was 41.4, 53.9, and 67.2% for PCV7, PCV10, and PCV13 serotypes, respectively, among all age groups; for patients < 2, 2–5, and > 60 years of age, PCV7 coverage was 50, 51, and 35%, respectively, PCV10 coverage was 53, 74, and 45%, respectively, and PCV13 coverage was 63, 80, and 68%, respectively (Hanna-Wakim et al., 2012). In countries that introduced PCV-10 or PCV-13 on their immunization schedules, an increase related to non-vaccine serotypes (NVTs) has been observed (Balsells et al., 2017). To date, reported increases in NVTs in the MENA are largely lacking. In Europe and the Western Pacific regions, but not in North America, serotype 24F has been reported as one of the emerging NVTs (Balsells et al., 2017). In France, a rebound in pneumococcal meningitis due to the strong emergence of serotype 24F, frequently penicillin-resistant, was observed in a 16 year French nationwide population-based study, especially during 2015 and 2016 (Ouldali et al., 2018). The 24F group serotype was found to be responsible for several instances of penicillin non-susceptible related IPD in Catalonia, Spain (Munoz-Almagro et al., 2011). In addition, it has been associated with macrolide-lincosamide and tetracycline resistance (Janoir et al., 2016). In Lebanon, LIPSP ongoing surveillance has recently revealed an increase in the number of 24F isolates, with 4 isolates in 2019.

Neither the available vaccines PPV or PCV nor the ones being developed (PCV15 and PCV20) covers the 24F serotype, therefore, it is crucial to monitor the susceptibility and epidemiology of this serotype. Thus, the current study aims to characterize *S. pneumoniae* serotype 24F isolates among IPD cases in Lebanon.

## Materials and Methods

### Isolate Collection

Eight *S. pneumoniae* isolates, belonging to serogroup 24, were collected among a total of 587 isolates through the Lebanese Inter-hospital Pneumococcal Surveillance program (LIPSIP), a prospective surveillance program, established in 2005 and still ongoing. The study included samples collected from 78 hospitals across Lebanon (Beirut, North and South Lebanon, Mount Lebanon and Bekaa) including patients of all age groups, diagnosed with IPD. When identifying pneumococcus from blood, CSF, or other sterile sites, hospitals alert the surveillance coordinator. Within 24 h, samples are collected by the courier, subcultured, and frozen after proper labeling and identification. Demographic data and clinical information were collected for all isolates. All the isolates were stored at −80°C till tested.

### Identification and Antibiotic Susceptibility Testing (Phenotypic Tests)

Bacterial isolates were cultured on Mueller-Hinton blood sheep agar (MHSB) plates and incubated at 37°C with 5% CO_2_ for 24 h. Identification of each isolate was done using colonies morphology, optochin susceptibility test and confirmed using matrix-assisted laser desorption ionization-time of flight mass spectrometry (MALDI-TOF MS) using MALDI Biotyper software (Brucker Daltonics, Bremen, Germany). Antibiotic susceptibility testing was processed according to VITEK^®^-2 platform manufacturer’s instructions. Briefly, a bacterial suspension using 0.45% sodium chloride solution was adjusted to an optical density of 0.5–0.63 McFarland units. The VITEK-2 AST-ST03 test card (bioMérieux, France) was inoculated with the bacterial suspension. The cards were loaded into the VITEK^®^-2 system and results were automatically reported by VITEK^®^-2 Software release 8.01. Antibiotic agents tested include: chloramphenicol (C), tetracycline (T), sulfamethoxazole-trimethoprim (SXT), vancomycin (Va), erythromycin (E), clindamycin (DA), levofloxacin (LVX), and oxacillin (Ox), as recommended by the Clinical and Laboratory Standards Institute (Clinical and Laboratory Standards Institute [CLSI], 2019). For oxacillin resistant isolates, E- tests were additionally performed for both penicillin (PEN) and ceftriaxone (CRO). For all isolates other than those from CSF, PEN susceptibility and resistance refer to PEN MIC of ≤ 2 and ≥ 8 μg/ml, respectively. Moreover, isolates with ceftriaxone (CRO) MIC ≤ 1 μg/mL were considered susceptible while those with MIC ≥ 4 were resistant. For CSF isolates, PEN susceptibility and resistance refer to PEN MIC of ≤ 0.06 and ≥ 0.12 μg/ml, respectively, while for CRO, MIC ≤ 0.5 μg/mL were considered susceptible while those with MIC ≥ 2 were resistant. In isolates that showed intermediate and/or resistant phenotype were defined as non-susceptible. At the molecular level, all isolates were species identified using the 16S rRNA gene sequencing useful to identify the *S. pneumoniae* based on the location of cytosine at the 203 position (Arbique et al., 2004).

### Whole-Genome Sequencing (WGS) and Annotation

NucleoSpin Microbial DNA kit (Macherey-Nagel, Germany) was used to extract the genomic DNA of the eight strains. The extracted DNA was then subjected to shearing using g-tubes (Covaris, United States). Genomic libraries were prepared according to the microbial multiplexing protocol according to the manufacture instructions without size selection. Sequel I platform (Pacific Biosciences, California, United States) was used for long reads sequencing. The Microbial Assembly pipeline featured in SMRT Link v8.0 was used to perform the assemblies of the genomes with the default minimum seed coverage (30X). ResFinder 3.2 (Zankari et al., 2012), CARD (Alcock et al., 2020), PlasmidFinder (Carattoli et al., 2014), VirulenceFinder 2.0 (Kleinheinz et al., 2014), and VFDB (Liu et al., 2019), ISfinder database, and MLST 2.0 (Larsen et al., 2012) were used to detect antibiotic resistance genes, plasmid replicon type, virulence genes, mobile elements and multilocus sequence types (STs), respectively. RAST 2.0 combined with BLASTP/BLASTN were used to predict open reading frame (ORF) (Brettin et al., 2015). Mauve v.2.3.1 was used to perform comparative genome alignments while diagrams and gene organization were sketched using Inkscape 0.92.4.

### Molecular Characterization of Their Capsular Genes

A multiplex-PCR assay was performed to detect isolates’ serotypes according to the Center for Disease Control’s protocol. Briefly, seven multiplex PCR reactions were run including 38 primer pairs, each pair corresponding to a specific serotype^1^. DNA of CDC *S. pneumoniae* isolates of known serotypes were used as positive controls. PCR reactions were run in Bio-Rad C1000^TM^ Thermal Cycler C1000, then the products were run on a 1% agarose gel. Finally, the obtained bands were compared to their corresponding positive controls.

Additionally, all the isolates were checked for 92 capsular genes (CR931632-CR931722, JF911515.1 and HV580364.1) (Bentley et al., 2006; Kapatai et al., 2016). FASTA files were downloaded from NCBI nucleotide database using BLAST^®^+ v.2.10.1 (Camacho et al., 2009). Serogroup/type 24F identification was performed through detection of associated genes with a cut-off of 80% coverage query and a 95% sequence identity (Kapatai et al., 2016).

### Multilocus Sequence Typing (MLST)

MLST was performed to determine the sequence type (ST) for all the *S. pneumoniae* strains by uploading the sequences to the PubMLST Database. Sequence types (STs) and assignment to clonal complex (CC) was performed using PHYLOViZ 2.0 program. STs sharing at least five allelic variants composed a CC (Mayanskiy et al., 2017).

### Phylogenetic Analysis

Forty-six *S. pneumoniae* genomes of serogroup 24 were downloaded from PubMLST as references representing 86 downloaded complete and draft genomes along with our eight complete genomes. These strains were phylogenetically clustered using core genome single-nucleotide polymorphisms (SNPs) by parsnp v1.2, available in the Harvest suite (Treangen et al., 2014) using the *S. pneumoniae* R6 (AE007317.1) as reference. SNPs identified in local collinear blocks were subsequently used for reconstructing an approximate maximum-likelihood tree using FastTree 2 (Price et al., 2010) while including the general time reversible (GTR) model of nucleotide substitution. The Shimodaira–Hasegawa test implemented in FastTree2 was used to assess the support for significant clustering in the observed phylogeny.

### Genotypic Antibiotic Resistance Profile

The isolates were analyzed for their penicillin-binding proteins (PBP) signature and GPSC number by uploading assembled genomes to Pathogenwatch^2^ where modifications of PBP to the native PBP1A, PBP2B, and PBP2X were also inspected. Antibiotic-resistance genes for all antibiotics were searched against a comprehensive antibiotic resistance database (CARD) using assembled genomes as input (Alcock et al., 2020).

### Virulence Gene Profile

WGS was used to detect virulence factors such as pneumococcal surface protein A (*pspA*), pneumococcal surface protein C *(pspC/cbpA)*, pneumococcal adherence and virulence factor A (*pavA and pavB*), cell wall hydrolytic enzymes including *lytA, lytB, lytC*, and *pce (lytD and cbpE*), neuraminidases A, B and C (*nanA*, *nanB*, and *nanC*, respectively), streptococcal enolase *(eno)*, pneumococcal choline binding protein A (*pcpA)*, pneumolysin (*ply*), choline binding proteins including *cbpD* and *cbpG* and pneumococcal serine-rich protein *(psrp)*, the metal-ion-binding proteins (*psaA, cppA, piaA, and piuA)* as well as zinc metalloproteases (*iga*, *zmpB*, and *zmpC*), high temperature requirement A (*htrA*) and capsule related genes (*cps*). We used the VFanalyzer, an automatic and comprehensive platform for accurate bacterial virulence factors identification, downloaded from the Virulence Factors database (VFDB).

### Ethical Considerations

Since data and samples from patients were collected prospectively through the IRB-approved LIPSP, and since the analysis of the bacteria was done in the absence of any patient identifiers, no additional ethical approval or informed consent from patients or guardians were required.

### Statistical Analysis

Collected data were coded, introduced, and entered to the software Statistical Package for Social Sciences (SPSS) version 25 (SPSSTM Inc., Chicago, IL United States). At first, descriptive analyses were performed using numbers and percentages for qualitative variables and means with standard deviation (SD) for continuous variables. Second, the univariate analyses were conducted, Fisher’s exact test was used for the comparison of percentages between two qualitative variables, in the event of an expected value *n* < 5. A logistic regression model was carried out to identify the association between the time periods (before 2013, 2013–2018, and 2019) and the prevalence of serogroup/serotype 24. The strength of association was interpreted using the odds ratio (OR) with 95% Confidence Interval (CI). A *p*-value < 0.05 was considered statistically significant.

## Results

### Patient Characteristics

We collected 8 isolates of serotype 24F among a total of 587 invasive *S. pneumoniae* isolates, through the LIPSP from 2013 to 2019. Our findings showed a significant time effect where there was a significant increase of the prevalence of serotype 24F observed over 3 different periods (period 1: before 2013, period 2: from 2013- to 2018, and period 3: 2019) with a *p*-value < 0.001 and OR = 8.837, 95% CI = (2.894–26.985). The isolates were mainly recovered from children less than 6 years old (87.5%). Males constituted 62.5% of the patients.

The source of the eight isolates were from the blood (75%, *n* = 6), followed by CSF (12.5%, *n* = 1), and pleural fluid (12.5%, *n* = 1). Four isolates were recovered in 2019, while the others were isolated in 2013, 2017, and 2018 (Table 1). Pneumococcal vaccination status information was lacking although PCV-13 was introduced to the national immunization program in January 2016 to be given in three doses at 4, 6, and 12–15 months.

**TABLE 1:** Clinical and demographic characteristics of the invasive pneumococcal isolates of serotype 24F in Lebanon.

Isolate	Clinical data							Serotype
	Year	Origin	Sex	Age (m/y)	Sample source	Diagnosis	Outcome	
310	2013	MGH-Beirut	Female	3 m	blood	Bacteremia	N/A	24F
475	2017	Haykel hospital- North LBN	Female	8 m	CSF	Meningitis	Death	24F
521	2017	Nini hospital- North LBN	Female	5 y	blood	Bacteremia	N/A	24F
525	2018	El Youssef hospital-North LBN	Male	44 y	pleural fluid	Pneumonia	N/A	24F
563	2019	Haykel hospital- North LBN	Male	6 y	blood	Pneumonia	Recovered	24F
566	2019	AUBMC-Beirut	Male	3 y	blood	Bacteremia	Recovered	24F
573	2019	MGH- Beirut	Male	2 y	blood	Pneumonia	N/A	24F
574	2019	MGH-Beirut	Male	5.5 y	blood	Meningitis	N/A	24F

*LBN, Lebanon; AUBMC, American University of Beirut Medical Center; MGH, Makassed General Hospital; m, month; CSF, cerebrospinal fluid; y, year; N/A, no available data.*

### Phenotypic Susceptibilities

Using the latest CLSI breakpoints, the susceptibilities of the *S. pneumoniae* strains against 10 antibiotics are summarized in Table 2. All the isolates were susceptible to penicillin except for 475 isolated from CSF and considered resistant. All of them were ceftriaxone, chloramphenicol, levofloxacin, and vancomycin sensitive. Resistance to tetracycline, erythromycin, clindamycin, and trimethoprim/sulfamethoxazole was 100% and 62.5, respectively.

**TABLE 2:** Comparison of phenotypic and WGS-derived antimicrobial resistance profiles of invasive Lebanese pneumococcal isolates of serotype 24F along with their MLST profiles.

Isolate	Phenotypic										Genotypic antibiotic resistance									MLST	GPSC
	MIC interpretation								*E*-test		Penicillin resistance				other antibiotics					ST	
	E	C	TE	Va	DA	LVX	SXT	Ox	PEN	CRO	PBP1a	PBP2b	PBP2x	ermB	tet(M)	rlmA(II)	pmrA	patB	patA		
310	R	S	R	S	R	S	S	R	0.5	≤ 0.12	17	15	22	+	+	+	+	+	+	11618	10
566	R	S	R	S	R	S	R	R	0.75	0.94	17	15	22	+	+	+	+	+	+		
574	R	S	R	S	R	S	R	R	0.25	0.094	17	15	22	+	+	+	+	+	+		
573	R	S	R	S	R	S	R	R	0.38	0.25	17	1	44	+	+	+	+	+	+	14184	
475	R	S	R	S	R	S	R	R	0.25	0.064	17	15	367	+	+	+	+	+	+	15253	
521	R	S	R	S	R	S	S	R	0.25	< 0.016	17	15	367	+	+	+	+	+	+		
525	R	S	R	S	R	S	S	R	0.5	0.064	17	15	367	+	+	+	+	+	+		
563	R	S	R	S	I	S	R	R	0.38	0.064	17	15	367	+	+	+	+	+	+		

*The isolates are sorted according to their STs. Abbreviations: MIC, minimum inhibitory concentration; S, susceptible; I, intermediate; R, resistant; N/A, no available data; MLST, Multilocus Sequences Typing; ST, sequence type; GPSC, global pneumococcal sequence clusters; E, erythromycin; C, chloramphenicol; TE, tetracycline; Va, vancomycin; DA, clindamycin; LVX, levofloxacin; SXT, Trimethoprim Sulfamethoxazole; OX, oxacillin; PEN, penicillin; CRO, ceftriaxone. “+” indicates the presence of the indicated genes.*

### Comparison of Phenotypic and Genotypic Antibiotic Resistance Profiles

Resistance to β-lactams in *S. pneumoniae* occurs mainly through mutations in the genes coding for the PBPs essential for the bacterial cell wall synthesis. BLAST results of the PBPs protein types and the eight genomes showed that three isolates (310, 563, and 574) had the 17 (PBP1a)–15 (PBP2b)–22 (PBP2x) signature explaining the penicillin susceptible phenotype predicted by the mode MIC (MM) model (Li et al., 2016), the other isolates showed new PBP protein type combinations 17 (PBP1a)–1 (PBP2b)–44 (PBP2x) (for the isolate 573) and 17 (PBP1a)–15 (PBP2b)–367 (PBP2x) (for the isolates 475, 521, 525, and 563) (Table 2).

Moreover, for isolates showing erythromycin-clindamycin resistance, blast results in CARD showed the presence of *erm(B)* gene potentially responsible for the phenotypic resistance in these cases.

The eight isolates harboring the *erm*(B) gene, also harbored the *tet*(M) gene (Alcock et al., 2020). Other genes such as *patA, patB, pmrA*, and *rlmA(II)* encoding drug resistance mechanisms through efflux pumps and alteration of the antibiotic targets, respectively, were also detected. All the isolates harboring the *erm*(B) gene or *tet*(M) also harbored the mobile elements Tn*6002* of the transposon Tn*916* family (Table 2; Calatayud et al., 2010; Treangen et al., 2014; Akdogan Kittana et al., 2019). Moreover, *tet*(M) and *erm*(B) were both carried on Integrative and conjugative element (ICE) ICESp12ST230 (Lo et al., 2020).

### Characterization of Pneumococcal Virulence Genes

To gain insight into genetic features promoting virulence, we investigated the presence of major pneumococcal-protein virulence factors (Table 3 and Supplementary Table S1). Interestingly, when comparing our isolates to other *S. pneumoniae* isolates belonging to CC230, they share similar virulence genes profile. Among the virulence factors, choline-binding proteins such as *cbpD*, *cbpG*, *lytA, lytB, lytC* and *pce/cbpE*, *pspA*, and *pspC/cbpA* were detected among all isolates in addition to *pavA* and *lmb* known as fibronectin and laminin-binding proteins, respectively. Hyaluronidase (*hysA*), *nanA* and streptococcal enolase (*eno*) as well as other genes known for their roles as iron and manganese uptake (*piaA, piuA*, and *psaA* and *cppA*, respectively), IgA1 protease (*iga*), *zmpB* and serine protease (*htrA*) were detected in all eight isolates, except for isolate 574 which lacks the *piaA* gene. For the capsule-related genes, *cpsA/B/C/D* were detected among all isolates. However, none of the isolates harbored *zmpC*, pilus-1 *(PI-1)* related genes, or pilus-2 (*PI-2*) genes. Other virulence factors of adherence properties such as rlrA islet were not detected among these strains except those belonging to CC162 and CC72 mainly found in United Kingdom.

**TABLE 3:** Virulence genes detected among Lebanese invasive pneumococcal isolates of serotype 24F.

Genes		Isolates							
		310	475	521	525	563	566	573	574
Adhesion	*cbpD*	+	+	+	+	+	+	+	+
	*cbpG*	+	+	+	+	+	+	+	+
	*lytA*	+	+	+	+	+	+	+	+
	*lytB*	+	+	+	+	+	+	+	+
	*lytC*	+	+	+	+	+	+	+	+
	*pce/cbpE*	+	+	+	+	+	+	+	+
	*pspA*	+	+	+	+	+	+	+	+
	*pspC/cbpA*	+	+	+	+	+	+	+	+
	*pavA*	+	+	+	+	+	+	+	+
Enzyme	*hysA*	+	+	+	+	+	+	+	+
	*nanA*	+	+	+	+	+	+	+	+
	*eno*	+	+	+	+	+	+	+	+
Anti-phagocytosis	*cps(A/B/B/D)*	+	+	+	+	+	+	+	+
Iron uptake	*piaA*	+	+	+	+	+	+	+	−
	*piuA*	+	+	+	+	+	+	+	+
Manganese uptake	*psaA*	+	+	+	+	+	+	+	+
Protease	*cppA*	+	+	+	+	+	+	+	+
	*iga*	+	+	+	+	+	+	+	+
	*htrA/degP*	+	+	+	+	+	+	+	+
	*tig/ropA*	+	+	+	+	+	+	+	+
	*zmpB*	+	+	+	+	+	+	+	+
Toxins	*ply*	+	+	+	+	+	+	+	+

*The present table shows the absence (-) or presence (+) of each considered genes from the VirulenceFactors database using VFanalyzer.*

### Phylogenetic Analysis

**FIGURE 1:**
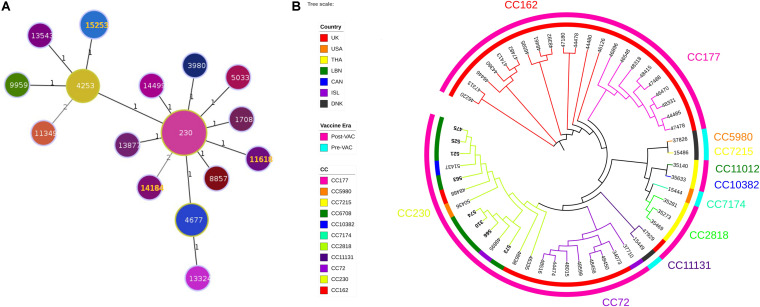
**(A)** Minimum Spanning Tree (MST) analysis of serogroup 24 isolates. PHYLOViZ full MST algorithm presenting clonal complexes (CCs) using sequence types (STs) belonging to serotype 24 deposited in the PubMLST database as of 20 June 2020. STs are represented by circles proportional in size to the number of isolates. STs highlighted in orange corresponds to the Lebanese study isolates. Branch labels are the number of allelic variations between STs (number 1 represents single locus variant while number 2 represents double locus variants). STs links are grayscaled where darker links have less differences than the lighter gray links. **(B)** Whole genome SNP-derived phylogenetic tree. SNP-based phylogeny of 54 genomes, including 8 from the present study and 46 publically available closed genomes from the PubMLST database. Colored numbers presented on the outer most perimeter represents the MLST clonal complex of the isolates starting from its corresponding branches. Inner circle indicates the geographic origin of each strain: red: United Kingdom, orange: United States of America, yellow: Thailand, green: Lebanon, dark blue: Canada: purple: Iceland and black: Denmark. Outer circle is the study periods where cyan and fuchsia represent the pre- and post-vaccination periods, respectively. The leaf labels indicate the PubMLST isolates ID of the selected *S. pneumoniae* isolates.

Three ST were identified (ST11618, ST14184, ST15253). Analysis of ST and assignment to clonal complexes (CCs) were performed using goeBURST (Figure 1A; Francisco et al., 2009). goeBURST full minimum spanning tree (MST) showing relationships among *S. pneumoniae* serotype 24 STs compared to serotype 24’s available in the global PubMLST database revealed that the ST11618 and ST14184 originated from ST230. ST11618 differs in one single allele (*aroE*) from ST230 while ST14184 differs in two alleles (*gdh* and *ddl*). ST15253 originated from ST4253 and differs also in two alleles (*aroE* and *spi*) from ST230. Population snapshot of 412 *S. pneumoniae* isolates of serogroup 24 using goeBURST full MST algorithm was added as Supplementary Figure S1.

Phylogenetic tree constructed from SNPs of representative 46 strains available in the global PubMLST database showed that among the 54 isolates, 11 CCs were identified (3 CCs PreVac, 6 CCs PostVac, 1 CC in both periods) (Figure 1B). The full data of these strains are presented in the Supplementary Table S2. The Lebanese isolates included in the study belonged to CC230 which emerged after PCV with three different STs. Isolates (475, 521, 525, and 563) having same ST15253 clustered and matched exactly with a Canadian isolate (PubMLST ID:51437). The other 3 isolates (566, 310, and 574) having same ST11618 clustered together with an isolate from Iceland (ST230); whereas, the isolate 573 having ST14184 did not cluster with any Lebanese isolate. Moreover, our isolates belonged to GPSC10, which is a common lineage of CC230 isolates.

## Discussion

The vaccine-related IPD incidence has dropped substantially by the introduction of PCVs. Nonetheless, non-PCV serotypes incidence is raising global concerns. So far, this is the first report in the Middle East and North Africa to describe the complete genome sequences of the emerging NVT 24F causing IPD in Lebanon. Serotype 24F isolates represent 1.36% of all the surveillance isolates. These isolates were mainly recovered from children less than 6 years of age during the period 2013–2019.

Among NVTs, serotype 24F is considered one of the most prevalent causative agents of IPD in Europe and the Western Pacific Region (Balsells et al., 2017; Kawabata et al., 2020). 24F, collected as part of the French national survey program of pneumococcal infections, showed the highest invasive disease potential during the PCV13 period in children < 2 years old (Varon et al., 2015). Furthermore, serotype 24F and other NVT such as 8, 12F, and 33F were considered at the upper end of the invasiveness spectrum among children immunized with PCV (Balsells et al., 2018).

Serotype 24F isolates share the same ST (or CC) with other serotypes as shown in MLST database. Serotype 24F belongs to the clonal complex CC230 among other serotypes including serotypes 14 and 19A which are also related to clone Denmark^14^-ST230. This lineage was reported to be largely distributed in southern part of Europe among children and adults in the PCV7 era (Aguiar et al., 2010). Likewise, 24F isolates belonging to CC156 with ST162, are correlated with 9V serotype and related to clone Spain^9V^-ST156 (Hanage et al., 2005; Sadowy et al., 2010; Kavalari et al., 2019). Both WGS and MLST typing confirmed that the Lebanese isolates were genetically homogeneous belonging to CC230 and clustered closely with isolates originating from Canada, United States, United Kingdom, and Iceland.

In β-lactam resistant *S. pneumoniae* isolates, the transpeptidase domains of PBPs were found to be altered thereby reducing their susceptibilities to cephalosporins and most penicillins (Li et al., 2016). Our 24F isolates had the 17-15-x signature, except for the isolate (573) with 17-1-44 signature. Interestingly, isolates having the 17-15-22 signature share the same ST 11618, while those having the 17-15-367 signature share ST15253 and the one with the 17-1-44 signature has the ST14184. Lower-level β-lactam resistance have been associated with mutations within PBP2b and PBP2x, along with, additional changes within PBP1a essential for high-level resistance. The diversification of *pbp* alleles is likely a consequence of transformation and homologous recombinational events among loci within *S. pneumoniae* and a number of closely related species resulting in a large pool of *pbp* alleles with diverse β-lactam MICs (Dewe et al., 2019).

Macrolides are among the first line-agents for the treatment of penicillin-resistant pneumococcal infections. Globally, macrolide resistance is geographically variable ranging from < 10 to > 90% of isolates (Schroeder and Stephens, 2016; El Moujaber et al., 2017). It is mainly driven via two mechanisms either by ribosomal dimethylation encoded by *erm*(B), efflux linked to the gene product of *mef* or due to mutations of the ribosomal target site of macrolides (Schroeder and Stephens, 2016). In this study, the *erm*(B) genes were present in all 24F isolates, whereas none of the isolates had *mef(*A) or *mef(*E) suggesting that macrolide resistance is mainly mediated by the *erm*(B) gene. *erm*(B) was the most detected macrolide resistance determinant (36%) among macrolide-resistant the strains followed by dual carriers of *erm* and *mef* genes (32%), and by *mef* carriers (18%) with 19F being the most prevalent resistant serotype (Taha et al., 2012). Similarly, in Europe, the *erm*(B) gene is predominantly detected in France (87.5%), Spain (77.3%), Switzerland (80%), and Poland (100%), whereas the *mef(*A) gene is more common in Greece (100%) and Germany (33.3%) (Reinert et al., 2005). Association of macrolide and tetracycline resistance was reported with the acquisition of *tet*(M) gene potentially through the conjugative transposon Tn*6002* frequently encountered in Europe (Calatayud et al., 2010; Treangen et al., 2014; Akdogan Kittana et al., 2019). Additionally, *tet*(M) and *erm*(B) were both carried on ICESp12ST230 (Lo et al., 2020).

Moreover, other genes were detected among our isolates such as *pmrA*, a pneumococcal multidrug resistance gene, coding for an efflux pump as well as two ABC transporters *patA* and *patB*. Overexpression of these genes was reported to be linked directly with decreased fluoroquinolones susceptibility in clinical isolates of *S. pneumoniae* (El Garch et al., 2010).

The polysaccharide capsule has been considered a major virulence determinant factor in *S. pneumoniae*. Other proteins have also contributed to pathogenesis and known to be involved in the disease progression mainly through their interactions with the host defense mechanisms (Gamez et al., 2018). Our 24F isolates carry a combination of virulence genes uniformly distributed among them; therefore, we did not find a correlation between virulence determinants distribution, antibiotic resistance genes and/or STs in our study. Finally, vaccines targeting non-serotype proteins are currently being studied as potential vaccine antigens. Among these, two were detected among our 24F isolates such the PspC and the PspA. These approaches aim to display broader protective immunity and to provide coverage against all circulating pneumococcal strains (Garcia-Suarez Mdel et al., 2006; Chan et al., 2019).

## Conclusion

In conclusion, we found a rise in serotype 24F among patients with IPD in Lebanon. This is the first report in the Middle East and North Africa region that characterizes the full genomes of *Streptococcus pneumoniae*, serotype 24F. The studied isolates, collected from different regions in Lebanon, revealed similar antimicrobial resistance profiles and genetically homogenous patterns Continuous surveillance is emphasized in order to characterize emerging NVT at both national and regional levels and help improve knowledge to expand the vaccine polyvalency.

## Data Availability Statement

The genomes of 310, 475, 521, 525, 563, 566, 573, and 574 have been deposited in GenBank under accession nos. CP046354, CP046355, CP046356, CP046357, CP046379, CP046358, CP046359, and CP046360, respectively.

## Author Contributions

GD and LR conceived and designed the study. IB, LR, JH, MF, and MM conducted the experiments. AZ, CB, GA, GM, TJ, MN, MH, MO, and GK collected the samples. IB, LR, MF, and GD analyzed the data and wrote the manuscript. All authors revised and approved the final draft.

## Conflict of Interest

The authors declare that the research was conducted in the absence of any commercial or financial relationships that could be construed as a potential conflict of interest.

## Footnotes

1 https://www.cdc.gov/streplab/downloads/pcr-us-clinical-specimens.pdf

2 https://pathogen.watch/

## Supplementary Material

Supplementary Figure 1 Population snapshot of 412 *S. pneumoniae* isolates of serogroup 24 using goeBURST full MST algorithm. STs that are linked by a line belong to the same cluster. Circle sizes are proportional to the number of isolates within the ST. STs links are grayscaled where darker links have less differences than the lighter gray links.

Supplementary Table 1 Virulence factors distribution among Lebanese invasive pneumococcal isolates of serotype 24F as well as 46 genomes available at the PubMLST database. VFanalyzer was used for the accurate bacterial virulence factors identification, downloaded from the VirulenceFactors database (VFDB: http://www.mgc.ac.cn/VFs/main.htm).

Supplementary Table 2 A complete list of the 54 genomes including 8 from the present study and 46 publicly available closed genomes from the PubMLST database. This table includes the ID, country, year of isolation, diagnosis, source, serotype, STs, CCs, and the GPSCs of the corresponding *S. pneumoniae* isolates.
