# Supplementary figures and images for: The Emergence of Invasive Streptococcus pneumoniae Serotype 24F in Lebanon: Complete Genome Sequencing Reveals High Virulence and Antimicrobial Resistance Characteristics
Source: Front Microbiol. 2021 Feb 19;12:637813. doi: 10.3389/fmicb.2021.637813 (PMC7967862; doi:10.3389/fmicb.2021.637813)

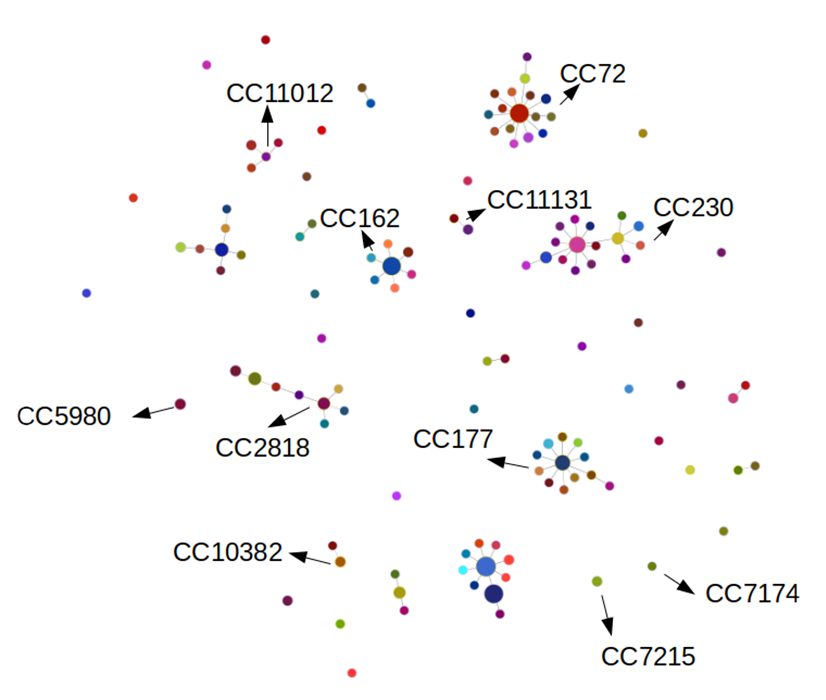

Supplement: Supplementary Figure 1 — Population snapshot of 412 S. pneumoniae isolates of serogroup 24 using goeBURST full MST algorithm. STs that are linked by a line belong to the same cluster. Circle sizes are proportional to the number of isolates within the ST. STs links are grayscaled where darker links have less differences than the lighter gray links. [file Image_1.png]
